# Ontogenetic and Environmental Variability of Hyssop (*Hyssopus officinalis* L.) Essential Oil Composition and Activity

**DOI:** 10.3390/plants15030487

**Published:** 2026-02-04

**Authors:** Renata Nurzyńska-Wierdak

**Affiliations:** Department of Vegetable and Herb Crops, Faculty of Horticulture and Landscape Architecture, University of Life Sciences in Lublin, 54 Doświadczalna Street, 20-280 Lublin, Poland; renata.nurzynska@up.lublin.pl

**Keywords:** Lamiaceae, bioactive substances, medicinal value, herbal therapies

## Abstract

Hyssop is an aromatic plant containing essential oil, used in folk medicine, and also known as a popular spice and ornamental plant. Hyssop essential oil is commonly used in cosmetics, perfumes, alcoholic and non-alcoholic beverages, and food additives. It can also be intended for external use as a fragrance ingredient in soaps, perfumes, creams, and other cosmetic products, as well as in aromatherapy. The composition of hyssop essential oil is not uniform and depends on a number of factors, including genetic, ontogenetic, and environmental ones. The hyssop essential oil is rich in oxygenated terpene compounds, the majority of which are represented by monoterpene ketones, i.e., isopinocamphone and pinocamphone. The essential oil yield ranged from 0.22% to 4.4% in different parts of the plant. The highest concentration of essential oil is found during full bloom. Annual plants accumulated the highest contents of volatile compounds, which was significantly influenced by genotype and year of cultivation. In addition, environmental conditions modify the composition of the essential oil of individual hyssop genotypes in different ways. Hyssop essential oil exhibits multi-faceted biological activities, depending on its chemical composition, which in turn depends on the stage of development and growing conditions.

## 1. Introduction

Herbs have been known for their potential to treat and prevent various ailments since ancient times. A growing interest in medicinal plants and herbal products has recently been observed in both developing and developed countries due to their efficacy and safety. Herbal medicine still underlies the primary healthcare for approximately 75% of the world’s population, especially in underdeveloped and developing countries. Herbal therapies are the most popular form of traditional medicine and are in high demand globally. Medicinal plants account for 80% of the raw materials used to produce medicines [1,2,3]. One example of such plants is hyssop, valued for its sedative, expectorant, and antitussive properties. This species has a long history of use in traditional medicine for stimulating digestion, improving circulation, and helping with various respiratory ailments, including coughs and sore throats. Furthermore, hyssop leaves are an aromatic spice that imparts a pungent flavor and minty aroma to dishes. In turn, hyssop nectar is used by bees to produce honey [4]. Its unique aroma is due to its essential oil, which is used in food and cosmetic production, as well as in aromatherapy. The biological value of hyssop essential oil depends on its composition, which varies as affected by multiple factors. It should be emphasized that studying individual variability factors is complicated by their mutual overlapping and modified mechanisms of action, especially that their effects can be positive, negative, or insignificant. The aim of this review article is to comprehensively present an assessment of the positive influence of ontogenetic and environmental factors on the chemical composition and biological activity of hyssop essential oil. It also analyzes the chemical composition of hyssop essential oils from various sources (different plant organs and collection regions) and their pharmacological activity, particularly in the context of current health issues.

## 2. Origin, Occurrence and Morphology of Hyssop

**Figure 1:**
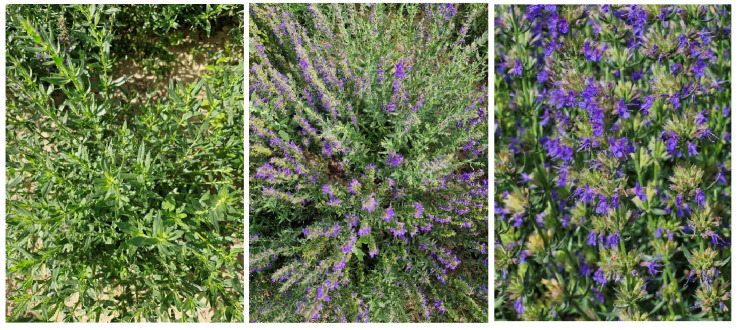
From the left: Hyssop plants in the vegetative and generative phases (photographs by the author).

The taxonomy of the genus *Hyssopus* L. is not yet fully established. It is typically assumed to comprise about 10–12 species, growing naturally in Europe, Asia, and northern Africa, and also introduced in Northern America. The most common representative of the genus is *Hyssopus officinalis* L., widely applied in traditional medicine, cuisine, and the fragrance industry [5]. It is a small, perennial subshrub belonging to the essential oil-rich family Lamiaceae. It occurs naturally in southern Europe, the Middle East and the Caspian Sea region. Its generic name comes from a misinterpretation of the Hebrew word *adobe* or *ezob*, namely, a biblical plant exhibiting laxative properties; it is also derived from the Arabic word *azzof*, meaning a holy herb [6,7,8]. Hyssop grows up to 30–70 cm in height and develops small, narrow, lancet-shaped, pointed and very fragrant leaves, as well as purple-dark blue, pink, white and sometimes red zygomorphic flowers gathered in a magnificent, peak inflorescence (Figure 1).

The flowering period spans from June to October. Colorful flowers attract bees, making hyssop a plant with high honey productivity (200–400 kg·ha^−1^). Within the species classified as *H. officinalis* L. ssp. *officinalis* Brig. var. *vulgaris* Benth., three botanical forms are distinguished, differing in flower colors: purple-blue—f. *cyaenus* Alefed; pink—f. *ruber* Alefed; and white—f. *albus* Alefed, which also differ in terms of the essential oil content and biological activity [8,9,10].

## 3. Hyssop Herbal Raw Material

Hyssop is an aromatic, essential oil-bearing plant providing valuable raw herbal material. Its leaves are used in folk medicine, represent a popular spice in European cuisine, and are also suitable for decorative purposes. The plant is listed in the official pharmacopeias of France, Portugal, Romania, Sweden, and Germany [5]. The above-ground parts of hyssop (hyssop herb—*Hysssopi herba*) are classified as an official medicine with expectorant, gastric, and diuretic indications. Hyssop is sourced from natural locations and also cultivated on herbal plantations in France, Italy, Germany, Serbia, Montenegro, Slovenia, Croatia, Bosnia and Herzegovina, Bulgaria, Hungary, the Netherlands, Finland, Poland, Moldova, Iran, China, India, Russia, and the USA. Its common cultivable varieties include Sophie, Erfurter Ysop, Blankyt, Cyrano, Domaći ljubičasti, and Perlay. Plants can be productive for about 10 years, and their above-ground parts are harvested during the flowering period [11,12]. The yield of fresh hyssop herb is 5–32 t·ha^−1^, that of its air-dried herb is 0.67–3.26 t·ha^−1^, and that of its essential oil can reach 10–20 kg·ha^−1^ [9,13]. Stancheva et al. [14] assessed the chemical composition of hyssop grown from seeds, propagated in vitro, and growing in natural habitats, and found the highest contents of antioxidant metabolites, phenols and flavonoids in flowers and leaves of the in vitro propagated plants. Extracts from *H. officinalis* leaves and flowers differed in their antioxidant potential, but the highest values were again found in the plants propagated in vitro. In turn, the highest concentration of essential oil was determined in the plants from natural habitats. Hyssop plants are primarily a source of essential oil, valuable for the cosmetics and perfume industry, especially for the production of oriental fragrances. Furthermore, hyssop essential oil exhibits antibacterial, antiviral, and expectorant properties. It is an important ingredient in aromatherapy, medicines, personal care products, food, and beverages [5]. Hyssop is also a valued spice, used in sauce recipes, as an ingredient of herbal mixtures (including the Middle Eastern za’atar mixture), and also to flavor liqueurs (it is one of the main ingredients of the official Chartreuse recipe) and honey [7]. The raw material available on the market often varies in terms of chemical composition and effects. Seeds are often mixed; hence, obtaining uniform plant populations for standardization purposes for the production of herbal teas, spices, ornamental flowers, honey feed for honey bees, and high-yield essential oil of uniform composition is quite difficult [15].

## 4. Hyssop Essential Oil Content and Composition

Hyssop essential oil is obtained mainly by steam distillation or hydrodistillation of the air-dried, flowering, above-ground parts of the plant. It is a clear, colorless or pale yellowish-to-green liquid with a herbal, camphor-like aroma, warm and spicy notes, and a pungent taste [9,16,17,18]. *Hyssopi herba* contains 0.3–1.8% of essential oil [9,17]. Previous studies have indicated a possibly higher content of essential oil (up to 7%) depending on the genotype [13]. The composition of hyssop essential oil is not uniform and depends on a number of factors, including genetic, ontogenetic, and environmental ones [9,12,18], as well as on the extraction method [19]. The hyssop essential oil is rich in oxygenated terpene compounds, the majority of which are represented by monoterpene ketones, i.e., isopinocamphone and pinocamphone. Other important components include monoterpene and sesquiterpene hydrocarbons, e.g., β-pinene (8.8%), germacrene D (5.4%), bicyclogermacrene (2.7%), and (E)-β-caryophyllene (2.6%), as well as oxygenated compounds, e.g., elemol (3.9%) and myrtenyl methyl ether (3.6%) [12,16,20,21]. Many other compounds of hyssop essential oil have also been identified (Table 1); the proportions of individual components of hyssop essential oil vary as influenced by, among others, ontogenetic and environmental factors.

## 5. Ontogenetic Variability

### 5.1. Plant Development Stages/Plant Organs

The chemical composition of hyssop essential oil depends on the type of distilled raw material (leaf, flower, herb) and the plant’s growth phase (pre-flowering, full flowering, post-flowering). Changes should be expected in the volatile oil content and composition during ontogeny. Both the flowering stages and plant parts have been shown to significantly affect the fresh and dry weight of the herb, dry leaf yield, and hyssop essential oil content [33]. Moghtader [26] found differences in the yield and chemical composition of the essential oil obtained from fresh leaves and flowers of *H. officinalis*. Thirty-five compounds were identified, accounting for 92.13% of the total oil obtained from leaves with a yield of 0.75% (*v*/*w*), and 36 compounds constituting 98.68% of the total oil obtained from flowers with a yield of 1.38% (*v*/*w*). The major components of the leaf essential oil were iso-pinocamphone (38.47%), pinocamphone (13.32%), n-decane (8.67%), and pinocarvone (5.34%), accounting for 40.25%, 14.92%, 8.63%, and 6.76% of the flower essential oil, respectively. Similarly, Pandey et al. [27] showed differences in the yield and chemical composition of the essential oil extracted from the leaves, flowers and stems of *H. officinalis* collected in India in the Western Himalayas region (Chamoli, Uttarakhand). The essential oil yield ranged from 0.22% to 4.4% in different parts of the plant. Furthermore, 57 components were identified, accounting for 99.8% of the leaf essential oil composition; 44 components accounting for 99.4% of the flower essential oil composition, and 57 components constituting 88.4% of the stem essential oil composition. The major components of the oils were cis-pinocamphone (49.7–57.7%), pinocarvone (5.5–24.9%), β-pinene (5.7–9.3%), 1,8-cineole (2.9–8.0%), β-phellandrene (1.8–3.2%), myrtenyl methyl ether (2.7–3.0%), sabinene (0.8–1.9%), isopimara-9(11),15-diene (<0.05–1.9%), myrtenol (1.4–1.7%), myrcene (0.5–1.3%), and trans-pinocamphone (<0.05–1.3%). The comparative analysis clearly indicated that the chemical composition of essential oils from leaves and stems was similar in terms of the content of cis-pinocamphone and pinocarvone, while the flower oil was distinguished by a higher concentration of pinocarvone.

Zawiślak [9] reported that trans-pinocamphone dominated in the essential oil of hyssop herb collected in the vegetative phase. Its content decreased with plant development, while the content of cis-pinocamphone increased. The β-pinene content was highest in the essential oil in the vegetative phase and more than half lower at the beginning and at full flowering. Can et al. [21] determined the yield and qualitative characteristics of hyssop depending on the plant development phase: before flowering, at the beginning of flowering, in the full flowering period, and during the post-flowering period. They determined the highest essential oil content (0.93%) and essential oil yield (24.01 L·ha^−1^) during the full flowering period. The major components of the analyzed essential oil were pinocamphone (38.41–41.85%), isopinocamphone (22.73–22.99%), and β-pinene (7.92–8.94%). The maximum pinocamphone content was found before flowering. Yousefzadeh and Naghdi Badi [34] determined that 0.459 to 0.618% of the essential oil is in the aerial parts of hyssop and reported the highest and lowest essential oil yields in the full flowering and pre-flowering stages, respectively. Finally, the essential oil yield obtained in the full flowering stage was five times higher than in the pre-flowering stage. In turn, Can et al. [21] demonstrated a significant effect of harvest time on the content and yield of hyssop essential oil, which were highest in the full flowering stage (0.93% and 24.01 L·ha^−1^, respectively). The major components of hyssop essential oil were pinocamphone (38.41–41.85%), isopinocamphone (22.73–22.99%), and β-pinene (7.92–8.94%), with the maximum pinocamphone content found before flowering. The study by Kotyuk [35] showed changes in the quantitative and qualitative composition of the essential oil of *H. officinalis* throughout the growing season. In the vegetative growth phase, 25 compounds were identified, the dominant ones being elemol (33.25%), germacrene D (21.59%), and bicyclogermakrene (15.78%). In the flowering phase, 30 compounds were identified, with high contents of isopinocampone and pinocampone (44.43% and 35.49%) and lower contents of myrtenol (5.26%), pulegone (2.93%), and bicyclogermacrene (1.35%). In turn, 21 compounds were identified in the fruiting phase, with elemol (44.46%), bicyclogermacrene (10.30%), D germacrene (5.86%), spatulenol (4.36%), β-eudesmol (4.34%), α-eudesmol (4.04%), and γ-eudesmol (3.92%) found to predominate. Overall, the essential oil from annual hyssop plants can be intended for use in the food industry, while the essential oil of plants harvested during the flowering phase is more suitable for use in cosmetic and perfume industries.

### 5.2. Daily and Seasonal Changes

Both the content and chemical composition of essential oils of certain plant species are subject to both diurnal and seasonal fluctuations. Kara and Baydar [31] obtained the highest essential oil content (0.57%) from hyssop flowers in full bloom and the highest essential oil yield (9.2 kg·ha^−1^) from hyssop herb in full bloom. Furthermore, the highest essential oil concentration was determined in the evening for both fresh and dried hyssop herb (0.14 and 0.48%, respectively). The composition of the analyzed essential oil depended on raw material harvest time, i.e., decreased concentrations of myrcene, limonene, sabinene, and linalool as well as increased concentrations of neryl acetate, farnesene, geranyl acetate, cadinol, heneicosene, and camphor were determined in the hyssop essential oil as the harvest time progressed (from morning to evening). Khan et al. [32] conducted a study on the seasonal variability of the composition of hyssop essential oil from March to October. They showed pinocamphone to be the major component of hyssop oil, with its content increasing successively in the post-flowering phase. The content of the second main component, β-pinene, initially increased and subsequently decreased after the full flowering phase. To sum up, the harvest period has a significant impact on the quantity and quality of hyssop essential oil. This finding indicates the importance of choosing the appropriate harvest time to obtain essential oil of the finest quality in the highest possible quantity for a specific purpose (production of medicines, cosmetics, and/or flavorings).

### 5.3. Plant Age

Plant age can also have a significant impact on the quantity and quality of essential oil, with a decreasing trend in older plantations. The oil yield of young hyssop plants in the pre-flowering phase is lower compared to older plants in the full flowering and post-flowering phases [32]. Németh-Zámbori et al. [36] found that annual hyssop plants accumulated more volatile compounds than two- and three-year-old plants (1.401%, 1.022%, and 0.880%, respectively), a finding also significantly influenced by genotype. Similarly, Kotyuk [35] reported that the content of essential oil decreased over the three-year life cycle of hyssop plants, decreasing by 1.007%, 0.75%, and 0.71% in the first, second, and third years, respectively. Even greater changes were noted in the essential oil composition. The volatile oil composition in plants in the first year of plant life included 46 components, with pinocampone (53.73%), isopinocampone (4.66%), myrtenol (9.35%), and camphor (3.86%) dominating. Thirty components were identified in the volatile oil of *H. officinalis* from the third year, with the following major components: isopinocampone (44.43%), pinocampone (35.49%), myrtenol (5.26%), germacrene D (3.15%), pulegone (2.93%), and bicyclogermacrene (1.35%). In contrast, Gille and Floria [37] demonstrated a significant increase in essential oil yield in a local population of the pink-flowering cultivar De Ciorani from the second to the third year and concluded it could be due to the most favorable meteorological conditions.

### 5.4. Intraspecific Diversity

**Figure 2:**
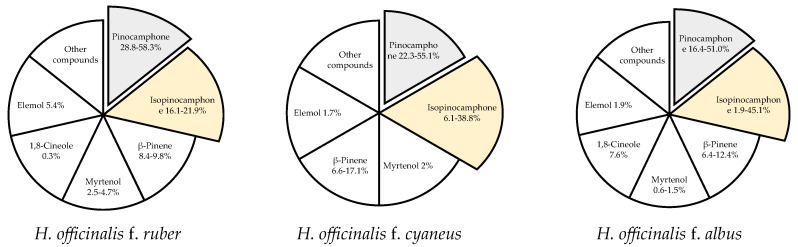
The dominant compounds of hyssop essential oil [6,38,39].

Changes in the chemical composition of the essential oil of *H. officinalis* may be triggered by many factors, with intraspecific differences being the most significant one. Hyssop contains more than 1% of essential oil, with the highest content recorded at the beginning of the flowering phase. The content and chemical composition of the essential oil also depend on the plant genotype. According to Jankovsky and Landa [13], the content of essential oil in 6 representatives of the phenotype (corolla color from dark blue to white) was determined by the color of the flowers (a higher content was found in individuals with a darker color), whereas stems without leaves and other parts of the plants did not contain essential oil. Likewise, Aćimović et al. [6] analyzed the content and chemical composition of essential oil isolated from white-flowered (f. *albus*), pink (f. *ruber*), and purple-blue (f. *cyaneus*) hyssop and determined from 0.47% (f. *albus*) to 0.74% (f. *cyaneus*) of volatile oil. In turn, Mohamadpoor et al. [38] reported smaller differences in the essential oil content in the inflorescences of two Iranian varieties of *H. officinalis* spp. *angustifolius*, i.e., 0.40 and 0.45 mL·100 g^−1^ for white and purple flowers, respectively. Intraspecific variability also applies to the chemical composition of hyssop essential oil (Figure 2).

Among the 59 identified compounds, the main one found in all genotypes was pinocamphone in its cis and trans forms, i.e., trans-pinocamphone in f. *albus* (16.4%) < f. *cyaneus* (22.3%) < f. *ruber* (58.3%) and cis-pinocamphone in f. *ruber* (16.1%) < f. *cyaneus* (38.8%) < f. *albus* (45.1%). The total content of these two compounds in all analyzed genotypes exceeded 60% (61.1–74.4% depending on the genotype). Analysis of essential oils isolated from the inflorescences of two Iranian varieties of *H. officinalis* spp. *angustifolius* with white and purple flowers showed 25 and 22 components (accounting for 98% of the total essential oils, respectively) [38]. The main components of the essential oil from the purple variety were cis-pinocamphone (55.14%), β-pinene (17.06%), and trans-pinocamphone (3.50%), while camphor (31.85%), cis-pinocamphone (30.11%), β-pinene (12.26%), and trans-pinocamphone (6.09%) were the major components of the essential oil from the white hyssop variety. Baj et al. [39] evaluated the chemical composition and antimicrobial activity of essential oils extracted from *H. officinalis* with white and pink flowers. The major component of the essential oil of white-flowered plants was pinocamphone (51%), while equal contents of pinocamphone (28.8%) and isopinocamphone (21.9%) were determined in the essential oil of pink-flowered plants. The essential oil of the pink form was more active against Gram-positive bacteria, especially against *Bacillus subtilis*, than the essential oil of the white form. Furthermore, 61 compounds were detected in the essential oil of hyssop of the Domaći ljubičasti variety cultivated in Serbia [12]. The most abundantly represented were bicyclic monoterpene ketones: cis-pinokamphone (43.8%) and trans-pinokamphone (18.3%), accounting for 62.1% of total essential oil, followed by β-pinene (6.3%) and pinocarvone (6.1%). Other studies have indicated that the essential oil from air-dried leaves of *H. officinalis* collected in the Ajangbadi region (Nigeria) contained mainly monoterpene hydrocarbons, α-pinene (70.9%), and β-pinene (10.9%) [40]. The cluster analysis conducted to characterize and compare *H. officinalis* essential oil from Nigeria with other oils from different locations around the world revealed chemical variability of this species, encompassing at least eight different chemotypes. The compositional pattern of the Nigerian oil sample represents a different chemotype of *H. officinalis* essential oil than the previously described ones, with a dominant pinene fraction. These findings are very interesting due to the significant aromatic and therapeutic properties of α-pinene and β-pinene, compounds used as fungicides, flavors, fragrances, antivirals, and antimicrobials [41,42]. Németh-Zámbori et al. [36] compared five hyssop cultivars (German, Hungarian, and Polish) in terms of their development and essential oil production during a 3-year field cultivation period. The Hungarian cultivar Sophie produced the highest essential oil yield (up to 2.037 mL·100 g^−1^). Annual plants accumulated the highest contents of volatile compounds, which was significantly influenced by genotype and year of cultivation. Forty-seven components were identified in all essential oils, with cis- and trans-pinocamphone prevailing. The highest total content of these two components was determined in the German variety Erfurter Ysop (70.7%). The third main compound was β-pinene, which occurred in the highest proportions in the Hungarian varieties (11–19%).

## 6. Environmental Variability

Raw hyssop material is obtained from various sources (cultivation, natural habitats) and from plants growing in various environmental conditions. It has been found that hyssop can be cultivated in semi-arid climatic conditions in accordance with ISO standards [33]. The effectiveness of its cultivation in more difficult environmental conditions depends, among other things, on the variety and its adaptability. Galambosi et al. [11] reported that a Swiss hyssop variety, Perlay, exhibited poor growth and overwintering performance, with lower essential oil content and lower herb yields compared to local varieties. They also noticed that Swiss hyssop varieties were unsuitable for cultivation in the conditions of southern Finland, which require special cultivation techniques and the selection of varieties well adapted to these extreme climatic conditions (large daily and seasonal temperature variations, severe frosts, and a short growing season). Growing conditions and environmental factors can modify the content and chemical composition of hyssop essential oil. Results of the chemical analysis of essential oil of *H. officinalis* ssp. *officinalis* cultivated over three growing years indicated isopinocamphone as its most abundant component, the accumulation of which was negatively affected by temperature, while positively affected by precipitation. An opposite observation was made for the contents of pinocamphone and β-pinene [12]. Jahantigh et al. [30] assessed the composition of hyssop essential oil under salt stress conditions, including five salinity levels: 0, 2, 4, 6, and 8 dSm^−1^. With increasing salinity, the content of the essential oil extracted from the aerial parts during the flowering phase increased and reached the maximum value at EC 6 dSm^−1^. The main components identified in the control group and in plants exposed to salt stress were cis-pinocamphone, β-pinene, β-phellandrene, pinocarvone, myrtenol, elemol, myrcene, linalool, and germacrene D. The compounds of hyssop essential oil were found to be sensitive to environmental changes (salt stress), with more noticeable changes observed in the levels of the major oil components: cis-pinocamphone, β-pinene, β-phellandrene, pinocarvone, elemol, myrtenol, germacrene D, linalool, and myrcene. Differences in their contents could be due to the induction of specific enzymes involved in their biosynthesis by salt stress.

The evaluation of *H. officinalis* L. genotypes of the *ruber*, *cyaneus* and *albus* forms from Moldova under drought conditions showed that higher values of the indicators of quantitative traits directly affect productivity for the *ruber* and *cyaneus* forms compared to the *albus* form [43]. The essential oil content differed across the forms: f. *ruber*—2.53%, f. *cyaneus*—1.88%, and f. *albus*—1.43% of dry weight. The main compounds of the analyzed oils were pinocamphone in trans (-) and cis forms (for f. *cyaneus*, 51.77% cis (-) pinocamphone and 6.70% trans (-) iso pinocamphone; for f. *ruber,* 66.94% pinocamphone, 33.31% trans (-) iso- pinocamphone and 33.63% cis (-) pinocamphone; for f. *albus,* 61.1% trans (-) iso- and 2.15% cis (-) pinocamphone) for all genotypes, followed by β-pinene (8.49% for f. *cyaneus*, 7.38% for f. *albus*, and 4.15% for f. *ruber*) and β-phellandrene (from 3.64% for f. *ruber* to 6.79% for f. *albus*). The remaining essential oil compounds were found in lower and various concentrations, and some of them occurred only in one genotype. The qualitative and quantitative chemical composition of hyssop essential oil may be closely related to its antibacterial and medicinal properties. The composition of the essential oil extracted by hydrodistillation from the aerial parts of *H. officinalis* from Egypt included 33 compounds, with the major ones being cis-pinocamphone (26.85%), β-pinene (20.43%), trans-pinocamphone (15.97%), α-elemol (7.96%), durenol (3.11%), β-phellandrene (2.41%), caryophyllene (2.34%), (E)-2,6-dimethyl-1,3,5,7-octatetraene (2.27%), 3(10)-carene-4-ol, acetoacetic acid ester (2.14%), bicyclogermakrene (1.83%), myrtenol (1.73%), germacrene D (1.68%), limonene (1.56%), γ-eudesmol (1.36%), and linalool (1.08%) [28]. It can therefore be assumed that environmental conditions modify the composition of the essential oil of individual hyssop genotypes in different ways.

*Agrotechnical Factors*

The active substances produced by medicinal and aromatic plants are extremely sensitive to the agrotechnical practices used, including the number of cultivated plants, fertilization, and irrigation, which are the main factors determining production and quality. The availability of nutrients to plants, nitrogen in particular, supports the development of young leaves, which most often translates into a higher essential oil content compared to mature leaves, and has a significant impact on the chemical composition of essential oils. Nitrogen and phosphorus are key elements that support plant photosynthesis as well as the synthesis of primary and secondary metabolites in plants. This, in turn, contributes to the ceaseless production of terpenoids [44]. Toaima [4] reported that hyssop essential oil consisted mainly of pinocamphone (31.61–57.63%) and α-pinene (20.47–49.88%) and that its composition could be modified by cultivation conditions. Hyssop essential oil contains more than 5% of pinocamphone, compared to any other oil, which is responsible for its flavor and aroma. Cultivation in a limited space (30 cm row spacing) under high NPK fertilization improved the oil quality, increasing the pinocamphone content more than other treatments. Plants grown at smaller density (50 cm row spacing) with any fertilization level had less pinocamphone than the other plants. Ghanbari-Odivi et al. [44] demonstrated differences in the content and yield of hyssop essential oil and its chemical composition under the influence of varied organic fertilization. The essential oil content ranged from 0.98% to 1.45% for treatments with a high concentration of poultry manure and a medium one of cow manure, respectively, which differed significantly from the control treatment (1.17%). The essential oil yield obtained from manure-fertilized plants was 47.5–53.8 kg·ha^−1^, which was 42.5–61.6% higher compared to the control treatment. Plants fertilized with low and high concentrations of poultry manure, as well as the control plants, had high concentrations of cis-sabinene hydrate, cis-pinocamphone, elemol, β-eudesmol, γ-eudesmol, germacrene D, bicyclogermacrene, and (Z)-β-ocimene. In turn, the plants treated with a medium level of poultry manure, a medium level of sheep manure, and a high level of cattle and sheep manure had increased contents of sabinene, linalool, and, to some extent, α-thujene, compared to the plants from the other treatments. In the group of plants amended with a medium level of cattle manure, as well as low levels of cattle and sheep manure, analyses showed higher percentages of β-eudesmol, myrtenol, limonene, β-bourbonene, allo-aromadendrene, (E)-caryophyllene, α-pinene, camphene, and β-pinene. The content and chemical composition of the essential oil of aromatic plants can also be positively affected by irrigation (Marino et al., 2019 [45], Sałata et al., 2020 [46]). It has been confirmed by Moro et al. (2011) [47], who showed a higher content of essential oil with higher concentrations of inocamphone and iso-pinocamphone as well as β-bourbonene and β-pinene in irrigated hyssop plants compared to the non-irrigated ones.

## 7. Hyssop Essential Oil Activity

**Figure 3:**
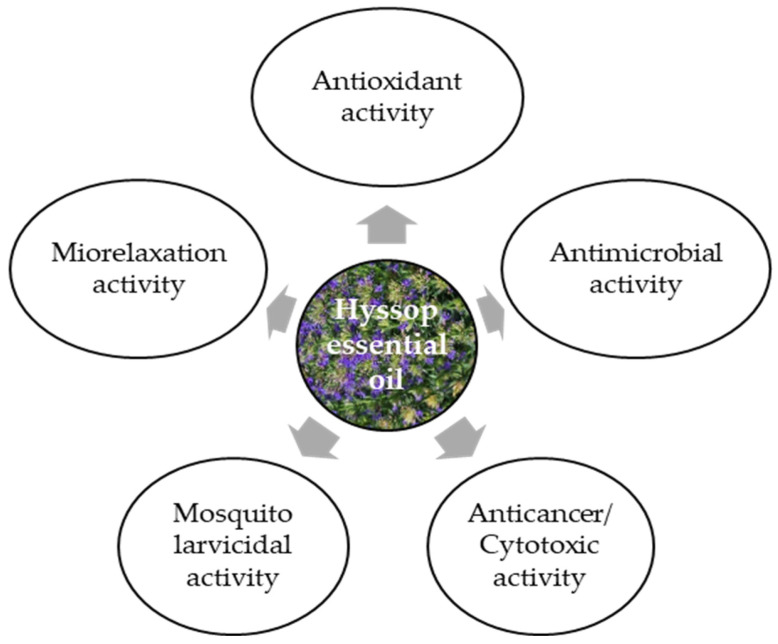
Biological activity of hyssop essential oil [10,15,24,38].

Hyssop essential oil is commonly used in cosmetics, perfumes, alcoholic and non-alcoholic beverages, and food additives. It can also be intended for external use as a fragrance ingredient in soaps, perfumes, creams, and other cosmetic products, as well as in aromatherapy. It is also an ingredient of bath salts, compresses, body oils, and massage oils. There are patented hyssop-based cosmetic preparations: JP 2004262861 of 24 September 2004, which is used against wrinkles, and KR2005073080 of 7 March 2015, which is used to treat acne, specifically against the *Propionibacterium acnes* bacterium [17]. The yield of hyssop essential oil ranges from 10 to 20 kg·ha^−1^, and varies greatly depending on the raw material type [15]. Hyssop essential oil can be used as a natural supplement in combating microbiological foodborne diseases, as well as an ingredient in flavorings (herbal, camphor scent with warm and spicy notes), especially in meat products, sauces, soups and spices [12]. Hyssop essential oil can be applied as a natural supplement in combating foodborne diseases of microbiological origin and as a component of flavor combinations (herbal, camphoraceous aroma with warm and spicy notes), especially in meat products, sauces, soups, and seasonings [12]. It exhibits multiple biological activities (Figure 3), depending on its chemical composition, which is in turn affected by the plant’s growth and development conditions and/or the type of raw material subjected to the distillation process.

The results of a study by Imbrea et al. [48] have indicated significant differences in the chemical profile of hyssop, with the species and cultivation site influencing its biological activity. They also showed that the physical and chemical soil conditions were more important for hyssop productivity than the climatic conditions. Local microclimate and soil properties can elicit minor changes, but they do not alter the basic chemical composition of the varieties. Hyssop essential oil exhibits moderate in vitro antibacterial activity as well as antifungal, insecticidal, and antiviral properties, and also excellent antioxidative and anticarcinogenic effects [15]. Its antioxidative properties are due to the presence of pinocamphone, iso-pinocamphone, β-pinene, and camphor [38]. Finally, the biological activity of hyssop essential oil can be attributed primarily to the dominant compounds but probably also to the synergistic effect of many secondary components. This may be a link between the ontogenetic and environmental variability of hyssop and the chemical composition of the essential oil and the resulting biological activity.

### 7.1. Antioxidant Activity

The chemical composition of essential oils can play a role in the antioxidative properties of plants, with key compounds having a particular influence. This characteristic is not determined solely by the concentration of a single compound, since major and minor components can act synergistically, enhancing the antioxidative properties [49,50,51,52]. The assessment of the antioxidative potential of hyssop essential oil is not unequivocal, as its antioxidative activity increases along with oil concentration, enhancing its antioxidative capacity [53]. Stan (Tudora) et al. [54] demonstrated very high activity of the essential oil from the hyssop variety Cătălin, which is distinguished by a significant proportion of monoterpenes (34.6% of cis-pinocamphone, 11.7% of trans-pinocamphone, and 10.5% of β-pinene). Antioxidative activity was also demonstrated for the hyssop essential oil containing camphor (23.61%) and β-pinene (21.91%) as its major components. Its total phenolic content was 23.16 mg of gallic acid per gram of essential oil, and its half-maximal inhibitory concentration (IC50) was 11.22 μg·mL^−1^ [10]. Kizil et al. [24] pointed out that the antioxidative activity of *H. officinalis* essential oil was relatively low and lower compared to that of butylated hydroxytoluene (BHT) and ascorbic acid. In addition, they emphasized that even small amounts of hyssop essential oil show detectable antioxidative activity. Likewise, Baj et al. [39] reported that the oil isolated from hyssop was characterized by low antioxidative activity with a half-maximal effective concentration (EC50) of 21.13 mg·mL^−1^ and a Trolox equivalent of 0.029 mM. In contrast, the results of agrotechnical studies indicated that hyssop plants fertilized with high doses of manure could produce an essential oil rich in myrtenol, cis-sabinene hydrate, and (Z)-β-ocimene, exhibiting strong antioxidative properties [44]. Ultimately, the differences in the antioxidative properties of hyssop essential oil likely result from its variable chemical composition, which in turn is an outcome of genetic factors, environmental conditions, and geographical origin. Hyssop essential oil has been proved to exhibit antioxidative properties and, thus, may be exploited in the production of cosmetics and pharmaceuticals, as well as in therapeutic interventions.

### 7.2. Antimicrobial Activity

Essential oils and their components have the potential to be used as natural antimicrobial compounds in pharmacy and food preservation and as alternatives to synthetic antibiotics in combating bacterial resistance [52]. Hyssop essential oil was shown to exhibit moderate and/or weak antibacterial activity against Gram-positive and Gram-negative bacteria and yeasts (Table 2), and lower activity compared to vancomycin, ciprofloxacin, and fluconazole [39].

On the other hand, a study by Kizil et al. [24] conducted with 5 and 10 μL samples of hyssop oil proved it exhibited strong antimicrobial activity against *S. pyogenes*, *S. aureus*, *C. albicans*, and *E. coli*, but not against *P. aeruginosa*. The diameter of the inhibition zones induced by 5 μL of the oil was smaller compared to the 10 μL sample, indicating a concentration-dependent effect. The antimicrobial activity of the essential oil might be closely related to its chemical composition and the presence of iso-pinocamphone. Based on minimum inhibitory concentration (MIC) data, Eldeghedy et al. [53] reported that *H. officinalis* essential oil showed low activity against *E. coli* ATCC 35218 and *S. aureus* ATCC 25923 (MIC: 80 μL·mL^−1^), and moderate activity against *P. vulgaris* ATCC 13315 (MIC: 20 μL·mL^−1^) and *S. aureus* (resis.) (MIC: 30 μL·mL^−1^). At the same time, the selected antibiotics showed varied activity against the tested bacterial strains; i.e., vancomycin at a dose of 30 μg showed activity against *S. aureus* ATCC 25923 (inhibition zone: 15 mm), whereas it was inactive against *S. aureus* (res.). Hristova et al. [23] showed that hyssop essential oil exhibited antifungal activity against 52 clinical isolates and reference strains of Candida spp. (Table 2). Its activity was stronger compared to pure cis- and trans-pinocamphone, α- and β-pinene, and β-phellandrene, and it inhibited the growth of both fluconazole-susceptible and fluconazole-resistant strains.

The use of essential oils to control phytopathogens in organic farming can be promising. However, although their action is prompt, their effectiveness is limited due to their relatively rapid volatilization. Stan (Tudora) et al. [16] demonstrated the bacteriostatic (but not bactericidal) effect of hyssop essential oil on the phytopathogenic bacterium—*Pseudomonas marginalis*. Another study by this research group [54] confirmed antimicrobial activity of the essential oil obtained from the new Romanian hyssop variety ‘Cătălin’, whose major components included cis-pinocamphone (34.63%), trans-pinocamphone (11.72%), β-pinene (10.5%), germacrene D (7.3%), and terpinene (7.2%). No inhibitory effect of the oil on the growth of the phytopathogenic bacterium *P. syringae* LMG5090 was shown, nor was any fungicidal effect demonstrated in the case of *Fusarium oxysporum*. In contrast, the analyzed essential oil exhibited a fungistatic effect and was able to delay mycelial growth and the extent of its inhibition depended on the concentration used.

The major components of hyssop oil, such as linalool, 1,8-cineole, methyleugenol, pinocamphone, isopinocamphone, β-pinene, and pinocarvone, contribute to its antimicrobial effect, both individually and synergistically with other essential oil components. The findings from the above-mentioned studies indicate the potential of hyssop essential oil to be used as an antimicrobial substance and a natural antioxidant in the pharmaceutical and food industries. It should be noted, however, that essential oils from *H. officinalis* exhibit moderate antimicrobial activity (dependent on their chemical composition), but they may be useful when a milder antimicrobial effect is preferred. Furthermore, the antimicrobial potential of hyssop essential oil can be utilized in the production of self-preserving cosmetic preparations [17].

### 7.3. Anti-Inflammatory Activity

Inflammation is a serious response of the live tissue to any type of injury and can be acute or chronic. Acute inflammation can be the body’s initial response to harmful stimuli. In chronic inflammation, the inflammatory response is disproportionate, causing damage to the body. Plants have enormous potential for producing new agents to be used in the treatment of chronic diseases [55,56,57]. In order to determine the potential anti-inflammatory properties of hyssop essential oil, Eldeghedy et al. [53] investigated its effect on the production of nitric oxide (NO) in RAW 264.7 cells (a murine macrophage-like cell line derived from a tumor induced by the Abelson murine leukemia virus, used as a model for research on, among others, the immune system or inflammatory conditions). NO production increases when an inflammatory stimulus begins, which blocks the pro-inflammatory effect. However, an increase in NO concentration in cells can be harmful and induce various inflammatory diseases. The application of hyssop essential oil at a concentration of 25 μg·mL^−1^ resulted in a 42.3% inhibition of NO release. These findings show that hyssop essential oil can be considered a promising phytotherapeutic agent due to its anti-inflammatory effects. It is noteworthy that data on the therapeutic efficacy and long-term side effects of essential oils as anti-inflammatory drugs are insufficient due to limitations in conducting clinical trials [58]. Further research on these valuable substances is necessary to allow for definitive conclusions regarding their potential application.

### 7.4. Cytotoxicity Effects/Anticancer Activity

The essential oils of many aromatic plants, as well as some of their components, exhibit anticarcinogenic effects in vitro and in vivo [59]. The essential oil of *H. officinalis* subsp. *aristatus* showed anticancer activity in the MTT assay against SW480 colon cancer, MDA-MB 231 breast cancer, and HeLa cervical cancer cell lines, as well as antigenotoxic activity [60]. Eldeghedy et al. [53] investigated the cytotoxic potential of hyssop essential oil on several human cancer cell lines at a concentration of 100 μg·mL^−1^, using dimethyl sulfoxide (DMSO) as a control, doxorubicin as a positive control, and cancer cells in a DMSO-free environment as a negative control. The essential oil of *H. officinalis* inhibited the growth of 100% of colon cancer cells HCT116, which made it a potent inhibitor of HCT116 growth. The effect of hyssop essential oil on prostate cancer and lung cancer cells was weak (inhibition of 17.2% and 21.5%, respectively) and moderate against pancreatic cancer, epidermoid cancer, and breast cancer cell lines (45.3%, 35.2%, and 33.2%, respectively). This oil is rich in the pinocamphone group compounds, i.e., bicyclic monoterpenes containing a ketone group. The main natural precursor of pinocamphone is β-pinene, which produces pinocamphone and its isomer isopinocamphone through oxidation or microbiological conversion, with trans-pinocamphone also being a key intermediate or related product in these biotransformations. Alpha- and β-pinene have been found to exhibit strong and multi-faceted biological activity [61,62], including inhibition of the development of breast cancer and leukemia [42]. The anticarcinogenic activity of hyssop oil, like other essential oils, is only the first element on the road to its potential use as a drug. Pharmaceutical studies conducted with both cell cultures and animal models, as well as biotechnological research, are, however, necessary to develop an efficient and safe therapeutic agent.

### 7.5. Other Types of Activities

Furthermore, hyssop essential oil exerts potential antispasmodic and antiplatelet effects. Mazzanti et al. [63] reported that it inhibited contractions induced by acetylcholine and BaCl2, with IC50 values of 37 μg·mL^−1^ and 60 μg·mL^−1^, respectively. Hyssop essential oil has also been found to exert a myorelaxant effect on isolated intestinal muscle preparations of guinea pigs and rabbits [64]. This relaxant effect is believed to be due to isopinocamphone, although synergistic actions are not excluded. The essential oil and isopinocamphone inhibited contractions induced by acetylcholine and BaCl_2_ in the guinea pig ileum in a concentration-dependent manner (IC50 42.4 and 61.9 μg·mL^−1^ for acetylcholine; 48.3 and 70.4 μg·mL^−1^ for BaCl_2_). It has been suggested that hyssop essential oil may elicit a valuable myorelaxant effect in antispasmodic drugs. Furthermore, it was tested for its antiplatelet effect and inhibition of clot retraction in the plasma of guinea pigs and rats. As noted by Tognolini et al. [65], the phenylpropanoid moiety was a favorable chemical feature for inhibiting platelet aggregation, and the absence of this moiety in hyssop essential oil underlies its inactivity.

Hyssop essential oil also exhibits insecticidal activity and can be used in the production of bioherbicides. Benelli et al. [66] analyzed the activity of essential oils in terms of their mosquitocidal action as potentially effective and environmentally friendly tools for combating vectors from the Culicidae family. *Culex quinquefasciatus* Say (Diptera: Culicidae) is a vector of lymphatic filariasis and dangerous arboviral diseases, such as West Nile encephalitis and Saint Louis encephalitis. The essential oil of *H. officinalis* subsp. *aristatus*, which contained a high amount of the insecticide linalool (47.7%), showed low activity (median lethal dose = LD50 99.5 μL·L^−1^), because this monoterpene alcohol is not very effective as a larvicide against *C. quinquefasciatus* (LD50 247 mg·L^−1^). These authors added that the insecticidal activity of an essential oil does not always depend on its main molecules but reflects the interaction between all its components and the physiology and behavior of the insects. Furthermore, they suggested that the larvicidal efficacy can be enhanced by preparing simple, bi-component mixtures of essential oils, as effective, inexpensive, and environmentally friendly mosquito larvicides. Tudora et al. [29] demonstrated the potential allelopathic effect of ‘Cătălin’ hyssop essential oil on the germination of weed and vegetable seeds. The analyzed oil exhibited an allelopathic effect, inhibiting/stimulating seed germination and subsequent seedling development. However, the concentrations showing an inhibitory/stimulatory effect varied depending on the seed species tested.

## 8. Conclusions

Hyssop essential oil possesses various bioactive components, and factors such as geographical location, variety, growth conditions, and extraction methods significantly influence its bioactive profile. Changes in the content and chemical composition of hyssop essential oil, like other aromatic plants, have their own rhythm and dynamics. Environmental factors can accelerate or delay the plant’s transition through successive developmental phases, but they do not affect the fundamental relationships between morphological traits and the level of active substances in a given organ.

Harvesting the aerial parts (*Hyssopi herba*) at the beginning of full flowering ensures a high yield of essential oil and its favorable bioactive profile. High levels of pinenes and pinocamphone in trans (-) iso and cis (-) forms in hyssop essential oil, i.e., compounds exhibiting multi-faceted biological activities (antioxidative, antimicrobial, anti-inflammatory, antispasmodic, anticarcinogenic), can be obtained through proper selection of variety and agrotechnical measures. In addition, hyssop essential oil fosters therapeutic potential. Owing to its aromatic, antioxidative, anti-inflammatory, and antimicrobial properties, it finds application in the cosmetic and food industries. Its antimicrobial and insecticidal potential are also of greatest importance in agriculture. Further research should be conducted to determine the relationship between the chemical profile of hyssop essential oil and its biological activity, and subsequently the possibility of safe use in therapy.

## Figures and Tables

**Table 1:** Main chemical components of hyssop essential oil [9,10,12,19,20,21,22,23,24,25,26,27,28,29,30,31,32].

Component	**Group**	Chemical Formula	Content (%)
cis-Pinocamphone	monoterpenoids	C_10_H_16_O	4.68–65.40
Sabinene	monoterpenes	C_10_H_16_	28.4–57.2%
trans-Pinocamphone	monoterpenoids	C_10_H_16_O	1.30–53.0
Heneikozene	saturated hydrocarbons	CH_3_(CH_2_)_19_CH_3_	9.49–37.0%
Pinocarvone	monoterpenoids	C_10_H_14_O	0.44–29.17
Geranyl acetate	monoterpenoids	C_12_H_20_O_2_	7.5–25.0%
Camphor	terpenoids	C_10_H_16_O	0.8–23.60
β-Pinene	monoterpenes	C_10_H_16_	6.30–21.90
Elemol	sesquiterpenoid alcohols	C_15_H_26_O	0.55–17.21
1,8-Cineole	monoterpenes	C_10_H_18_O	0.47–14.25
Myrcene	monoterpenes	C_10_H_16_	0.90–10.5
β-Phellandrene	monoterpenes	C_10_H_16_	2.40–9.51
n-Decane	alkanes	C_10_H_22_	8.67
Farnesene	sesquiterpenes	C_15_H_24_	1.3–7.5
β-Phellandrene	monoterpenes	C_10_H_16_	2.40–7.50
Terpinen-4-ol	terpenes	C_10_H_18_O	1.00–7.13
Germacrene D	sesquiterpenes	C_15_H_24_	0.17–6.20
Sabinene	monoterpenes	C_10_H_16_	0.8–5.20
α-Pinene	monoterpenes	C_10_H_16_	0.30–4.09
Myrtenol	monoterpene alcohols	C_10_H_16_O	1.39–3.70
Limonene	monoterpenes	C_10_H_16_	0.60–7.19
Myrtenyl methyl ether	monoterpenoids	C_11_H_18_O	2.70–3.60
Durenol	phenolic compounds	C_10_H_14_O	3.11
Carvacrol	monoterpenoid phenols	C_10_H_14_O	3.02
Bicyclohepta.2-n	cyclic hydrocarbons	C_7_H_8_	2.98
Spathulenol	sesquiterpene alcohols	C_15_H_24_O	0.20–2.80
p-Cymene	aromatic hydrocarbons	C_10_H_14_	2.81
Bicyclogermacrene	sesquiterpenes	C_15_H_24_	1.53–2.70
Myrtenyl acetate	monoterpenoid esters	C_12_H_18_O_2_	0.98–2.61
(E)-β-Caryophyllene	sesquiterpenes	C_15_H_24_	0.98–2.60
Myrtenal	monoterpenoids	C_10_H_14_O	2.32

**Table 2:** Antibacterial properties of hyssop essential oil [10,12,23,39].

Bacteria	MIC *	MBC
Gram−		
*Escherichia coli* ATCC 8739	56.81 μL·mL^−1^	56.81 μL·mL^−1^
*Escherichia coli* ATCC 10536	56.81 μL·mL^−1^	113.63 μL·mL^−1^
*Escherichia coli* ATCC 25922	5 mg·mL^−1^; 156.25 μL·mL^−1^	5−10 mg·mL^−1^; 312.5 μL·mL^−1^
*Proteus hauseri* ATCC 13315	56.81 μL·mL^−1^	113.63 μL·mL^−1^
*Proteus mirabilis* ATCC 12453	5 mg·mL^−1^	10 mg·mL^−1^
*Klebsiella pneumoniae* ATCC 13883	5 mg·mL^−1^	10 mg·mL^−1^
*Pseudomonas aeruginosa* ATCC 9027	5 mg·mL^−1^	10 mg·mL^−1^
*Salmonella Enteritidis* ATCC 13076	113.63 μL·mL^−1^	113.63 μL·mL^−1^
**Gram+**		
*Staphylococcus aureus* ATCC 25923	28.40 μL·mL^−1^	56.81 μL·mL^−1^
*Staphylococcus aureus* ATCC 25923	5−10 mg·mL^−1^	10−20 mg·mL^−1^
*Staphylococcus epidermidis* ATCC12228	2.5−5.0 mg·mL^−1^	5−10 mg·mL^−1^
*Micrococcus luteus* ATCC 10240	2.5 mg·mL^−1^	5.0 mg·mL^−1^
*Bacillus cereus* ATCC 11778	56.81 μL·mL^−1^	56.81 μL·mL^−1^
*Bacillus subtilis* ATCC 6633	0.625−5.0 mg·mL^−1^	2.5−5.0 mg·mL^−1^
*Streptococcus pyogenes* ATCC 19615	0.312−0.625 mg·mL^−1^	0.625−1.25 mg·mL^−1^
*Streptococcus pneumoniae* ATCC 49619	0.312−0.625 mg·mL^−1^	0.625−1.25 mg·mL^−1^
*Streptococcus mutans* ATCC 25175	0.625−1.25 mg·mL^−1^	1.25 mg·mL^−1^
*Listeria monocytogenes* ATCC 19111	56.81 μL·mL^−1^	113.63 μL·mL^−1^
*Listeria monocytogenes* ATCC 19117	312.5 μL·mL^−1^	625 μL·mL^−1^
*Rhodococcus equi* ATCC 6939	56.81 μL·mL^−1^	113.25 μL·mL^−1^
*Listeria ivanovii* ATCC 19119	56.81 μL·mL^−1^	113.63 μL·mL^−1^
*Enterococcus faecalis* ATCC 29212	113.63 μL·mL^−1^	227.25 μL·mL^−1^
*Listeria innocua* ATCC 33090	113.63 μL·mL^−1^	227.25 μL·mL^−1^
*Bacillus spizizenii* ATCC 6633	113.63 μL·mL^−1^	227.25 μL·mL^−1^
**Yeast**	**MIC**	**MFC**
*Candida albicans* ATCC 102231	0.625 mg·mL^−1^	2.5 mg·mL^−1^
*Candida parapsilosis* ATCC 22019	0.625−1.25 mg·mL^−1^	1.25−5.0 mg·mL^−1^
*Candida glabrata* ATCC 90030	512−1024 μg·mL^−1^	1024−2048 μg·mL^−1^
*C. tropicalis* NBIMCC 23	512−1024 μg·mL^−1^	512−1024 μg·mL^−1^
*C. parapsilosis* ATCC 22019	256−512 μg·mL^−1^	512−1024 μg·mL^−1^
*C. krusei*	128−256 μg·mL^−1^	256−512 μg·mL^−1^

* Explanations: MIC: Minimum Inhibitory Concentration; MBC: Minimum Bactericidal Concentration; MFC: Minimal Fungicidal Concentration; the range of numerical data concerns white- and pink-flowered *H. officinalis* L.

## Data Availability

No new data were created or analyzed in this study. Data sharing is not applicable to this article.
